# Utility of a self-assembling peptide in the management of refractory hemorrhagic duodenal ulcers

**DOI:** 10.1055/a-2408-8600

**Published:** 2024-09-25

**Authors:** Takehide Fukuchi, Kingo Hirasawa, Shinpei Kondo, Shigeru Iwase, Shin Maeda

**Affiliations:** 1 36993 Department of Gastroenterology, Fujisawa City Hospital, Fujisawa, Japan; 2 Division of Endoscopy, Yokohama City University Medical Center, Yokohama, Japan; 3 26438 Department of Gastroenterology, Yokohama City University School of Medicine Graduate School of Medicine, Yokohama, Japan

Hemoclips, injection therapy, and thermocoagulation are the most commonly used methods of endoscopic hemostasis for controlling nonvariceal gastrointestinal bleeding. However, endoscopic hemostasis of hemorrhagic duodenal ulcers in high-risk patients remains difficult and may lead to bleeding-related deaths. Repeated thermocoagulation treatments may result in delayed perforation and the need for surgical intervention 1 2 . PuraStat (3-D Matrix, Tokyo, Japan) is a novel self-assembling peptide hydrogel developed as a hemostatic agent, which is believed to be effective for primary hemostasis. Furthermore, it is expected to reduce the risk of delayed perforation due to excessive cautery burns and has a wound healing effect 3 4 5 . Here, we describe a high-risk case in which PuraStat was used for a refractory hemorrhagic duodenal ulcer ( Video 1 ).

Utility of a self-assembling peptide in the management of refractory hemorrhagic duodenal ulcers.Video 1

A 78-year-old woman presented with an infected pancreatic pseudocyst associated with severe acute pancreatitis, which was resistant to antibiotics. The patient had hypoalbuminemia and chronic kidney disease, and was taking antiplatelet drugs for cerebral infarction. During her first hemorrhage, primary hemostasis was achieved with thermocoagulation; however, rebleeding occurred 2 days later and the patient underwent repeat thermocoagulation. She experienced rebleeding for the third time 5 days later and thermocoagulation was not performed due to the risk of delayed perforation. PuraStat (3 mL) was applied several times while pressing the catheter against the bleeding site, and hemostasis was achieved in approximately 2 minutes ( Fig. 1 ). Thereafter, no rebleeding was observed, the bottom of the ulcer became epithelialized, and scarring was observed 1 month later ( Fig. 2 ).

**Fig. 1:**
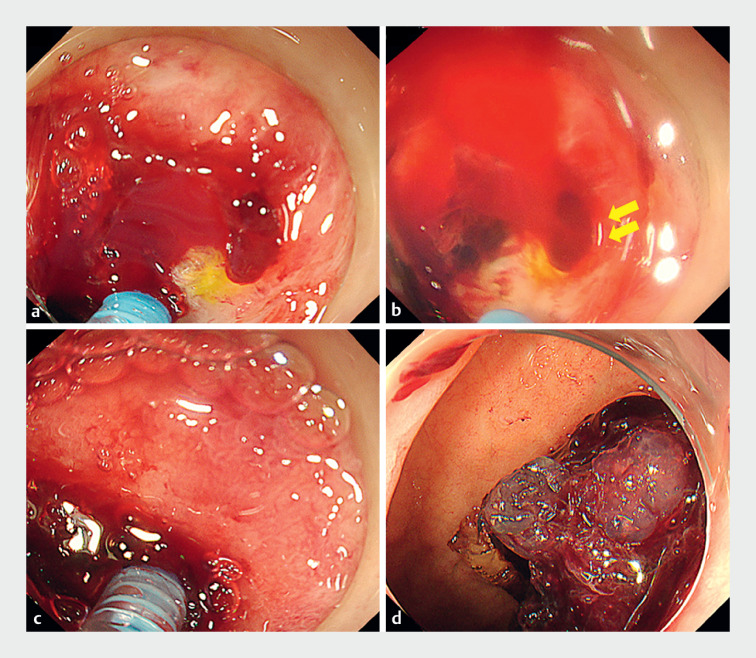
Endoscopy images. **a** The bleeding point was difficult to identify and the ulcer appeared to be deep. **b** PuraStat (3-D Matrix, Tokyo, Japan) application allowed simultaneous visualization (yellow arrows) and hemostasis by gel immersion technique. **c** PuraStat (3 mL) was applied several times while pressing the catheter against the bleeding site. **d** The gel ball appearance, shaped like a “bunch of grapes.”

**Fig. 2:**
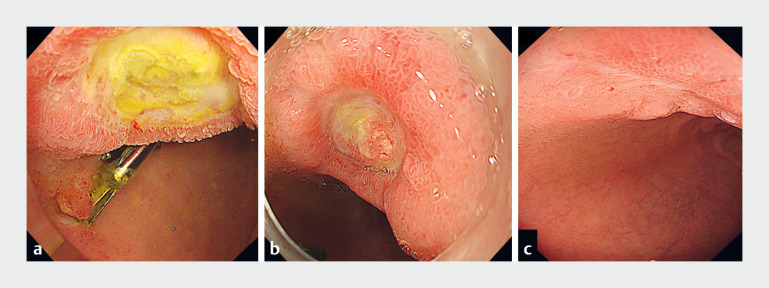
The bottom of the ulcer became epithelialized after hemostasis with PuraStat (3-D Matrix, Tokyo, Japan). **a** Endoscopic image 2 days after PuraStat application. **b** Endoscopic image 1 week after using PuraStat. **c** Scarring was observed 1 month later.

For refractory duodenal ulcers in high-risk patients, excessive thermal damage may not only cause delayed perforation but also prolong wound healing and increase the risk of rebleeding. This case suggests the usefulness of hemostasis using PuraStat, which does not require thermocoagulation.

Endoscopy_UCTN_Code_TTT_1AO_2AD

## Notes

Endoscopy E-Videos https://eref.thieme.de/e-videos
*E-Videos* is an open access online section of the journal *Endoscopy* , reporting on interesting cases and new techniques in gastroenterological endoscopy. All papers include a high-quality video and are published with a Creative Commons CC-BY license. Endoscopy E-Videos qualify for HINARI discounts and waivers and eligibility is automatically checked during the submission process. We grant 100% waivers to articles whose corresponding authors are based in Group A countries and 50% waivers to those who are based in Group B countries as classified by Research4Life (see: https://www.research4life.org/access/eligibility/ ). This section has its own submission website at https://mc.manuscriptcentral.com/e-videos .
